# From digestion to inflammatory signaling: the Postprandial Bioactive Exposure Axis as a compartment-based conceptual model for mechanistic nutrition research

**DOI:** 10.3389/fnut.2026.1859744

**Published:** 2026-10-16

**Authors:** Abdulmannan Fadel, Yazan Ranneh

**Affiliations:** 1 College of Medicine and Health Sciences, United Arab Emirates University, Al Ain, United Arab Emirates; 2 College of Pharmacy, Al Ain University, Al Ain, United Arab Emirates

**Keywords:** bioaccessibility, cardiometabolic inflammation, epithelial transport, food matrix, intestinal barrier, metabolic endotoxemia, postprandial metabolism

## Abstract

Mechanistic nutrition research often moves from what a meal contains to what it is claimed to do without clearly defining the exposure steps between ingestion and biological response. This becomes problematic when studies use causal or quasi-causal language about inflammation, barrier function or systemic effects. Digestion, intestinal interaction, epithelial uptake, enterocyte metabolism, microbial conversion, portal or lymphatic transport and post-absorptive handling can each alter the chemical form, concentration, timing and site of action of food-derived molecules. This article proposes the Postprandial Bioactive Exposure Axis, or PBEA, as a compartment-based model for organizing these steps in mechanistic nutrition research. The model separates four operational fractions: bioaccessible, intestinally bioactive, absorbed and systemically relevant. These fractions are levels of exposure inference, not discrete biological compartments. The aim is to help authors match claims to the compartment actually measured. The PBEA adds value by separating luminal availability from local intestinal action, and absorption from systemic relevance. It also helps prevent common overinterpretations, including equating intake with exposure, micellarisation with bioavailability, plasma detection with physiological relevance and indirect blood markers with direct evidence of altered barrier function. The model is intended as a practical tool for study design, model selection, temporal interpretation and more cautious mechanistic claims.

## Introduction

Mechanistic nutrition research often begins with a food, nutrient or named bioactive and ends with a claim about biological effect. That shortcut is weak when causal or quasi-causal language is used, because food-derived molecules pass through digestion, intestinal interaction, epithelial transfer, microbial conversion and post-absorptive handling before any downstream inflammatory response can be interpreted. Each step can change chemical identity, concentration, timing and site of action (1–3).

This problem matters most when nutrition studies move beyond association and begin to imply mechanism. A compound may be abundant in food yet poorly released from the matrix, released but poorly absorbed, absorbed mainly after transformation or detected in blood in a form that is not clearly relevant to a remote tissue (4–7). Interpretation errors can occur when these steps are not clearly defined. In such cases, interpretation may extend beyond what the data can reasonably support. In the current article, we introduce the Postprandial Bioactive Exposure Axis (PBEA) as a compartment-based way to organize this process. The PBEA is not intended to define a universal meal-to-inflammation pathway. Its purpose is more limited: to help track intermediate exposure steps and link interpretation to the compartment that was actually assessed. We use cardiometabolic nutrition as the primary example because postprandial lipemia, endothelial responses, barrier-related outcomes, endotoxin-associated markers, and low-grade inflammation are often discussed together in this field. This use is practical rather than restrictive. The PBEA could also be applied to other areas of mechanistic nutrition where digestion, transit, microbial metabolism, and systemic responses are interpreted within the same study.

## Conceptual synthesis approach

As this paper is presented as a conceptual analysis rather than a systematic review, the literature was not searched and selected systematically. This means that the synthesis is likely to be affected by selection bias and should not be read as a map of all available evidence. Instead, literature searches were continued until there was sufficient coverage of each primary transition in the PBEA sequence: digestion and bioaccessibility, intestinal epithelial interactions, apical-to-basolateral transport, post-absorptive metabolism, microbial conversion, and interpretation of human postprandial inflammatory responses. Recent primary studies, methodological papers, and human postprandial studies were prioritized when they helped illustrate uncertainty in one of these transitions, or when they showed where interpretation along a transition may become speculative. These included, but were not limited to, studies addressing assay specificity, metabolite identity, sampling time, and compartment mismatch (7–12). Evidence was then curated by reading each study according to the type of exposure claim it could support. This review does not attempt to combine effect sizes or directly compare dietary components. Instead, we aimed to define how far a mechanistic claim can reasonably extend based on the compartment that was directly measured.

### Why mechanistic nutrition research suffers from compartment confusion

The biological effect of a meal is not determined by food intake alone. It also depends on which molecules become available in each compartment, their concentrations, when they appear, and the local conditions they encounter. Composition, luminal release, epithelial contact, absorption, and systemic exposure are therefore connected, but they do not carry the same evidential weight. Treating these steps as equivalent can allow mechanistic conclusions to go beyond what the data support (6, 7, 10). Polyphenols provide a clear example. Parent compounds are still sometimes discussed as if they represent the main postprandial species in circulation. In practice, plasma and urine often contain conjugated, microbial or lower-molecular-weight metabolites rather than the intact compounds originally present in the food (5, 8). Inter-individual differences in gut microbial metabolism add another layer of variation to this exposure sequence (13). The same logic applies to lipid digestion products, peptides, lipoprotein-associated species and hepatically modified metabolites, whose biological interpretation depends on where and when they are measured (3, 10, 14).

### The Postprandial Bioactive Exposure Axis

The PBEA is a compartment-based interpretive model for organizing postprandial exposure from meal entry to downstream signaling. Figure 1 presents this sequence as four linked but non-identical fractions. The term “axis” is used here to describe an ordered sequence of interpretation, not a validated causal pathway. This order should not be read as a linear kinetic pathway, because gastric emptying, intestinal transit, bile delivery, gallbladder contraction, first-pass metabolism, lymphatic transport, tissue uptake and systemic clearance can alter the timing, concentration and chemical form of postprandial exposure. The model begins with digestion-generated luminal chemistry, then moves to local activity at the intestinal interface, net epithelial transfer into portal blood or lymph and finally remote tissue exposure in forms and amounts that are compatible with downstream signaling pathways (1, 8, 10).

**Figure 1:**
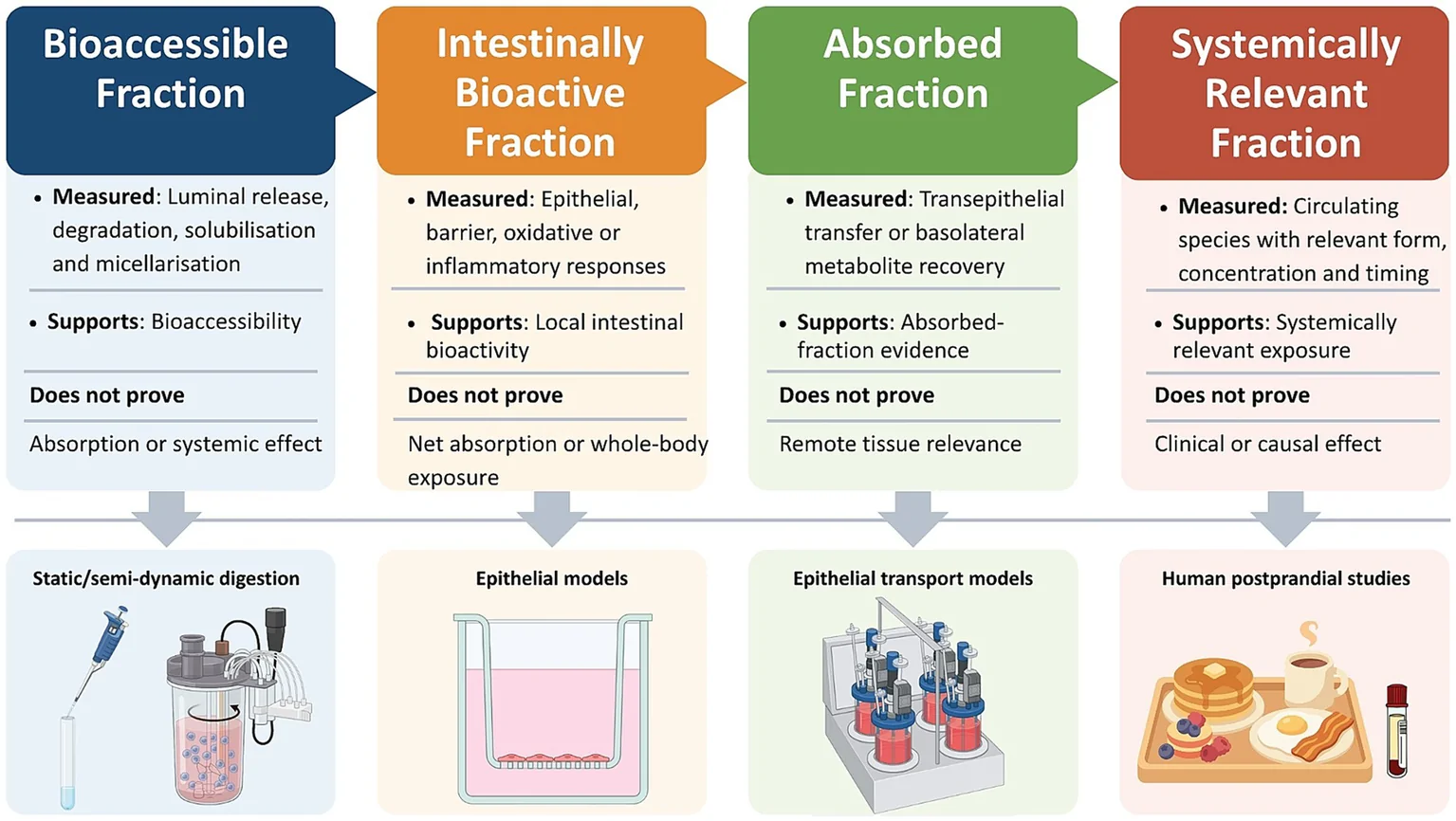
The Postprandial Bioactive Exposure Axis as a compartment-based model for interpreting postprandial exposure. The model separates four operational fractions: bioaccessible, intestinally bioactive, absorbed and systemically relevant. Each fraction supports a different level of inference. Movement from one fraction to the next requires additional evidence and should not be assumed from intake or detection alone.

### Operational fractions and what each one means

To make the model usable, four operational fractions are defined and summarized in Figure 1.

These categories are operational rather than absolute. They are intended to help authors state what has been measured and what remains unmeasured. In practice, these definitions are not always clearly separated. For example, a digestion-plus-cell-culture study may provide evidence relevant to both bioaccessibility and intestinal bioactivity. An epithelial transport study may support a claim about the absorbed fraction if basolateral recovery is measured, but it still does not establish whole-body exposure unless the transported species, concentration, and time course are linked to an appropriate *in vivo* context. Similarly, detecting a compound or metabolite in human plasma may support absorbed exposure or post-absorptive exposure, but it does not, on its own, demonstrate a tissue-level mechanism or clinical effect. When a single study provides evidence for more than one PBEA fraction, claims should be limited to the highest fraction that was directly measured, while the lower fractions can be used as supporting evidence.

### What the PBEA adds beyond existing terminology

The PBEA does not replace established terms such as bioaccessibility, absorption, bioavailability, biotransformation or systemic exposure. Its novelty is operational. It places these terms into an ordered postprandial sequence and links each step to the level of inference it can support. The PBEA is therefore not proposed as a new biological pathway or as a replacement for ADME concepts, but as a practical claim-boundary tool for postprandial mechanistic nutrition studies.

The PBEA has four practical uses. First, it separates luminal availability from local intestinal action. A digestion product can be bioaccessible without being intestinally bioactive, and it can be intestinally bioactive without being absorbed (1, 7). Second, it separates absorption from systemic relevance. For tissues protected by additional barriers, such as the blood-brain barrier, systemic exposure does not necessarily imply tissue access (45). A compound may cross an epithelial model or appear in plasma but still be present in a chemical form, concentration or time window that is unlikely to explain a remote biological effect (5, 8, 10). Third, it links model choice to claim boundaries. Digestion models, epithelial models, transport models, fermentation systems and human postprandial studies answer different exposure questions and should not be treated as interchangeable evidence (15–18). Fourth, it makes timing explicit, because early luminal release, epithelial transfer, lipoprotein handling and microbial catabolism do not occur at the same time (5, 8, 14).

The PBEA is therefore best understood as a tool for keeping claims close to the evidence. It does not propose a new biological pathway. It provides a structured way to decide whether a study supports a bioaccessibility claim, an intestinal bioactivity claim, an absorbed-fraction claim or a systemically relevant exposure claim.

### Digestion as the generator of the initial exposure field

The first exposure field is generated by digestion, not by the ingredient list. Standardized static and semi-dynamic INFOGEST workflows have made it easier to study how matrix structure, food processing and digestive conditions affect luminal release (7, 9). Static models are useful for this purpose and semi-dynamic methods help when gastric kinetics and staged delivery matter. Within the PBEA, gastric emptying and matrix disintegration are therefore treated as kinetic modifiers of the bioaccessible fraction. Structured matrices may generate early and delayed phases of luminal release as their physical structure progressively breaks down, meaning that gels, solid foods and emulsions of similar composition may produce different exposure profiles.

Even so, digestion models should be described for what they are: models of luminal transformation, not direct models of human bioavailability or disease risk (19).

This distinction matters because matrix effects are often large. Particle size, emulsification, protein-lipid interactions, fiber entrapment and thermal processing can all alter the release and physical presentation of food-derived molecules (20, 21). A compound that is present in a meal may remain poorly liberated. A compound that is liberated may enter mixed micelles or other supramolecular structures that alter its access to the epithelial surface (21). Digestion can also create locally relevant species that were not present in the intact food, including oxidized lipid products and new peptide pools (22).

The physical form in which a bioactive is presented after digestion can also influence subsequent exposure. Lipophilic compounds may partition into mixed micelles, while dietary proteins can bind bioactives or form surface coronas around food-derived particles, altering their stability and interaction with the epithelial interface. Protein or mucus-derived coronas can modify cellular uptake and transepithelial transport, while extracellular vesicles may protect encapsulated molecules during digestion and influence their delivery to epithelial or extra-intestinal tissues. Within the PBEA, these structures are therefore considered properties of the bioaccessible fraction rather than separate fractions, because they modify the form in which a compound is presented to the intestinal interface (21, 23–25).

For that reason, the bioaccessible fraction should be treated as the first experimentally defensible approximation of exposure in mechanistic nutrition studies. It is not the same as absorption, but it is usually a better starting point than intake alone.

### The intestinal interface as a regulated signaling site

The intestinal barrier is better understood as a dynamic mucosal interface than as a simple seal. It comprises mucus, epithelial cells and their junctional complexes and mucosal immune cells that continually survey and respond to luminal signals. Recent primary studies show that microbial activity can directly remodel gastrointestinal mucus, that tissue-resident immune cells can integrate within intestinal epithelium and survey the barrier and that human intestinal organoid-on-chip systems display stimulus-dependent epithelial responses rather than passive exclusion. In that sense, the barrier regulates selective interaction with the lumen rather than acting as an indiscriminate wall (26, 27).

Figure 2 illustrates the intestinal interface as a signaling site, not just as a wall. Luminal material can act locally without becoming systemically available. Cytokines, dietary components, microbial metabolites and digestion products can all influence junctional behavior, epithelial metabolism, mucus properties and immune signaling at this interface. That is the logic behind the intestinally bioactive fraction in the PBEA.

**Figure 2:**
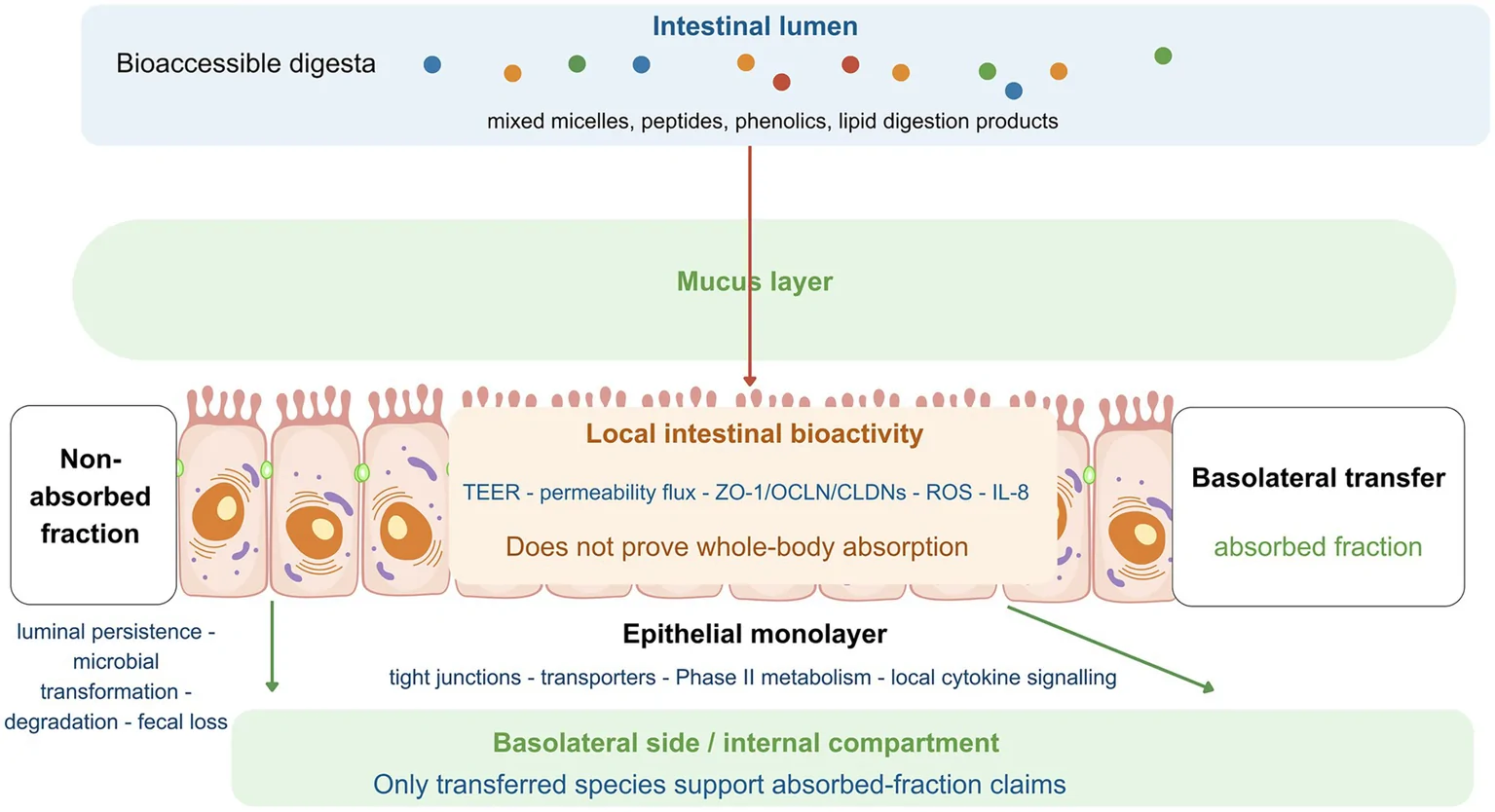
The intestinal interface as a checkpoint in the PBEA. Bioaccessible digestion products may interact with mucus, epithelial cells, transporters, tight junctions and local inflammatory signaling before any net absorption occurs. Epithelial endpoints such as TEER, permeability flux, oxidative stress, cytokine release and junctional markers support intestinal bioactivity, but they do not by themselves prove whole-body absorption or systemic relevance. The non-absorbed fraction is shown as a luminal pathway involving persistence, microbial transformation, degradation and fecal loss. Methodologically, intestinal permeability should not be inferred from any single readout. Dual-sugar tests such as lactulose-mannitol provide integrated *in vivo* recovery measures for small probe molecules and are useful for selected aspects of small-intestinal permeability, but they do not report permeability to larger luminal molecules and they do not by themselves localize the site or mechanism of barrier disturbance (29, 43). Junctional gene or protein measurements can support mechanistic interpretation, but barrier function depends on junctional organization and route selectivity, so expression changes are not equivalent to measured flux (44).

Commercial zonulin assays should be interpreted with care. Widely used ELISA kits have shown limited analytical specificity, and serum zonulin-family measurements have not correlated well with physiologic permeability assessed by lactulose-mannitol testing (12, 28). These methods may add context, but they should not be used as substitutes for one another (29, 30).

Barrier claims in nutrition research should therefore stay close to the measured data. Functional permeability tests, barrier structure assessments, and inflammatory markers each tell a part of the story, but no one test can confirm the entire mechanism.

### Transport, metabolism and temporal separation of exposure

Net absorption should not be equated simply with movement of material from the gut lumen into the bloodstream. Rather net absorption represents a continuum of processes including apical uptake, intracellular metabolism, efflux, basolateral transport and presystemic elimination. The absorbed fraction is thus not solely equivalent to the bioaccessible fraction transported across the epithelial barrier. The absorbed fraction is instead a chemically fractionated product of transport and metabolism processes (10, 18). Evidence of transepithelial transfer should therefore be interpreted as evidence of epithelial passage and presystemic handling rather than as a direct estimate of human absorption.

This distinction is especially relevant when the compounds ingested are not the same as the compounds later detected in circulation.

For polyphenols, plasma and urine commonly contain phase II conjugates and microbially derived catabolites rather than a simple reflection of the parent compounds present in food. The same logic applies more broadly to food-derived molecules subject to strong enterocyte, microbial and downstream metabolic processing. A study may still identify pathway sensitivity by dosing a parent compound, but it should distinguish this experimental signal from realistic postprandial exposure (5, 8).

Time is another neglected variable. Early postprandial sampling mainly reflects upper-gastrointestinal handling and rapidly appearing species, whereas later time points can capture lipoprotein remodeling, redistribution and microbial catabolite formation. A single blood sample therefore cannot represent the whole postprandial sequence (5, 8, 14).

### Microbial metabolism as an extension of postprandial exposure

The postprandial period does not end at the small intestine. For substrates that escape substantial small-intestinal absorption, colonic metabolism becomes a second stage of exposure shaped by the gut microbiota. Fermentation of fermentable fibers generates short-chain fatty acids, while incompletely absorbed (poly)phenols can be converted into smaller phenolic metabolites and catabolites that appear later in plasma and urine than the early small-intestinal phase. As a result, the molecules most relevant to late postprandial biology may differ markedly from those present earlier (5, 8, 31, 32). For this reason, the PBEA treats microbial metabolism as part of the exposure sequence rather than as a later, separate event. A vascular, immune or metabolic response that appears later in the postprandial period may be linked to microbial or host-microbial products, not necessarily to the parent dietary compound. Human metabotype studies also show that these later exposure patterns can differ between individuals, meaning that the same meal or polyphenol-rich food may lead to different circulating metabolite profiles (8, 13). This has two practical implications. Delayed effects should not be attributed automatically to the best-known parent compound. Study design should also account for the time separating small-intestinal handling from colonic transformation.

As a practical guide for human postprandial studies, the first 0–4 h after ingestion predominantly reflects upper-gastrointestinal absorption and early host metabolism, whereas metabolite appearance from approximately 8 h onward is more suggestive of colonic microbial transformation. The intervening 4–8 h period should be considered transitional rather than assigned to either compartment. These time windows are indicative rather than absolute because gastric emptying, small-intestinal transit and orocecal transit vary between meals and individuals. Where microbial metabolites are of interest, timing should therefore be interpreted together with metabolite identity, with extended plasma and/or urine sampling where feasible (5, 8).

### Endotoxemia as a compartment-sensitive example

Endotoxemia serves here as one example of compartment-sensitive interpretation, not as the main pathway proposed by the PBEA. It is a useful case because high-fat meal studies can sometimes move too quickly from postprandial lipid handling or endotoxin-related markers to broader claims about gut permeability, LPS translocation and inflammation. Human studies support biological plausibility in selected settings, especially when postprandial lipid handling, obesity or larger triglyceride excursions are present, but they do not establish endotoxin as a uniform or dominant mechanism of postprandial inflammation (33–35).

The human evidence is uneven for two main reasons. First, the response appears to depend on host phenotype and postprandial lipid handling. Controlled studies have reported greater changes in endotoxin-related markers and broader inflammatory or transcriptomic responses after high-fat exposure in adults with obesity or in those with larger postprandial triglyceride excursions (34, 35). Second, the analytical problem is substantial. The Limulus amebocyte lysate assay performs poorly in plasma unless masking and recovery issues are addressed, and endotoxin recovery can be strongly affected by plasma components and protein-endotoxin interactions (11). Within the PBEA, lipopolysaccharide-binding protein, soluble CD14 and LAL-based endotoxin measurements can support endotoxin-related response claims when assay limitations are addressed. They should not be treated as stand-alone evidence of increased gut permeability, direct LPS translocation or endotoxin as the dominant mechanism of postprandial inflammation (11, 33, 35).

### Model-to-compartment matching in experimental nutrition research

One practical use of the PBEA is model selection. Each model captures only part of the postprandial exposure sequence, so the endpoint should match the claim being made. Static and semi-dynamic digestion systems are best matched to the bioaccessible fraction because they describe luminal release, solubilization, degradation and matrix-dependent transformation rather than absorption itself. Semi-dynamic INFOGEST protocols can also change release kinetics and apparent bioaccessibility when gastric emptying and matrix structure are relevant (7, 9). When digestion outputs are transferred to epithelial models, digest conditioning should be treated as part of the experiment because it can affect epithelial compatibility and barrier preservation during Caco-2 exposure (36).

Epithelial systems such as Caco-2, Caco-2/HT29-MTX and organoid-derived monolayers should be interpreted according to their endpoint. TEER, permeability flux, junctional proteins, oxidative stress, cytokine release and transporter expression mainly support claims about intestinal bioactivity. Apical-to-basolateral transfer, basolateral metabolite recovery or transported-species identification support narrower claims about the absorbed fraction (10, 15–18). For direct exposure of Caco-2 monolayers to undigested extracts, we do not propose a numerical validity score because such weighting has not been experimentally validated. Instead, minimum validity requires a physiologically justified exposure concentration, appropriate viability and barrier-integrity controls, and either prior digestion or a clear justification for bypassing digestion. Without these conditions, responses to an undigested extract should be interpreted primarily as evidence of epithelial pathway sensitivity rather than intestinal bioactivity following consumption. Where mucus interaction is central to the hypothesis, a mucus-producing model should be considered. Fermentation models are best matched to delayed microbial transformation, while human meal studies are needed for integrated postprandial physiology. Even in human studies, interpretation depends on whether the sampling window and analytes match the expected biology, because early plasma conjugates, later microbial catabolites, lipoprotein remodeling and vascular responses do not peak together (5, 8, 14).

### Applying the PBEA in study design

In practice, the PBEA can be used as a pre-study checklist. Table 1 presents examples of common study designs that are often challenging to classify.

**Table 1:** PBEA-based interpretation of common study designs.

Study design or endpoint	PBEA classification	Supported claim	Unsupported claim
Static or semi-dynamic digestion only	Bioaccessible fraction	The food matrix generated a measurable luminal fraction after digestion	The food or compound was absorbed or had systemic activity
Digestion followed by epithelial exposure, with TEER, permeability flux, ROS, cytokines or junctional markers	Bioaccessible fraction plus intestinally bioactive fraction	The digest altered epithelial or barrier-related responses under the model conditions	The digest was bioavailable or produced a systemic anti-inflammatory effect
Epithelial transport study with basolateral recovery but limited chemical identification	Preliminary absorbed-fraction evidence	Some material crossed the epithelial model	The transported material is fully identified or relevant to tissue exposure
Epithelial transport study with basolateral metabolite identification and concentration data	Stronger absorbed-fraction evidence	Transported species and approximate exposure form were identified	The study proves clinical effect or remote tissue mechanism
*In vitro* colonic fermentation with metabolite profiling	Delayed microbial transformation	Microbial conversion generated secondary metabolites	The metabolites were absorbed or systemically active without transport or human data
Human postprandial plasma or urine metabolomics	Integrated absorbed or post-absorptive exposure	Circulating or excreted species, timing and inter-individual variation were described	The detected metabolites caused the biological effect
Human study using LBP, sCD14 or LAL-based endotoxin outputs only	Endotoxin-related response signal	The data support an endotoxin-related response if assay limitations were addressed	The meal directly increased gut permeability or caused LPS translocation
Human study measuring cytokines, CRP, vascular function or transcriptomics only	Integrated systemic response	A postprandial inflammatory, vascular or immune response occurred	The source compartment or causal bioactive species was identified

### Worked examples of PBEA-based interpretation

A polyphenol-rich food study provides a clear example. In conventional terminology, a study may state that a digested polyphenol-rich food has anti-inflammatory bioavailability because the digest reduces IL-8, oxidative stress or barrier disruption in Caco-2 or related epithelial models. Under the PBEA, the same study would be classified as evidence for a bioaccessible fraction plus intestinal bioactivity (7, 9, 15–17, 36). Without apical-to-basolateral recovery, transported-species identification or human plasma data, it would not support claims about absorbed exposure or systemic relevance (5, 8, 10, 14, 18).

A high-fat meal study provides a second example. In conventional terminology, a study may state that a high-fat meal increases gut permeability or causes endotoxemia because postprandial lipopolysaccharide-binding protein, soluble CD14 or LAL-based endotoxin outputs changed. Under the PBEA, postprandial triglycerides and chylomicron-related changes would support claims about lipid handling and postprandial lipemia, while lipopolysaccharide-binding protein, soluble CD14 or LAL-based endotoxin results would support only an endotoxin-related response signal if analytical recovery and assay limitations are addressed (11, 14, 33–35). Direct claims about increased gut permeability or LPS translocation would require additional evidence, such as validated permeability testing, assay recovery controls and temporally matched inflammatory, vascular or immune endpoints (12, 29, 34).

The same logic can also be applied to other dietary components. For complex polysaccharides and fermentable fibers, the PBEA separates luminal release, viscosity-related effects and local epithelial interaction from later microbial fermentation products such as short-chain fatty acids (37, 38). For micronutrients and carotenoids, it separates matrix release and micellarisation from epithelial uptake, transport and post-absorptive handling (23, 39). For intact proteins and digestion-derived peptides, it separates proteolytic release, local epithelial effects, basolateral transport and systemic peptide or amino acid exposure (40, 41).

A simple decision sequence can also be used when designing or interpreting a study. First, identify the measured compartment: luminal digest, epithelial response, basolateral transfer, microbial metabolite output, plasma or urine profile or whole-body endpoint. Second, define the chemical species measured, including parent compounds, digestion products, conjugates, lipoprotein-associated species or microbial metabolites. Third, does the timing of sampling match the expected biology? Fourth, limit the claim to the highest compartment for which there is direct data supporting it. Last, do not ascribe a systemic mechanism unless exposure, time, concentration and a biologically compatible endpoint were all measured.

In human postprandial studies, interpretation should also consider the magnitude and variability of the response. Effect sizes with confidence intervals should be reported alongside relevant kinetic measures, such as incremental area under the curve, maximum concentration and time to maximum concentration, rather than relying only on statistical significance at individual time points. Inter-individual variability should also be reported where relevant, particularly when differences in metabolic phenotype, postprandial response or microbial metabolism may influence the observed exposure profile.

## Discussion: how the PBEA improves inference

The PBEA improves interpretation by requiring authors to state where their evidence sits in the postprandial sequence. Its main value is not to re-label existing terminology, but to keep interpretation linked to the type of exposure that was actually measured. This helps address four common problems in mechanistic nutrition research: (1) assuming that intake equals exposure; (2) assuming that bioaccessibility equals absorption; (3) assuming that detection in plasma equates to physiological relevance; and (4) treating indirect blood markers as direct evidence of altered intestinal barrier function. These problems arise because food-derived molecules can be released, transformed, transported, conjugated, packaged into lipoproteins, metabolized by microbes or cleared before they reach a target tissue (5, 7, 8, 10). The model is particularly useful when a study combines digestion, epithelial models, postprandial plasma measurements and inflammatory endpoints, because the same experimental system can support different levels of inference depending on the endpoint used (Table 1).

The PBEA also helps separate stronger links from weaker ones. Digestion-dependent luminal transformation, stimulus-dependent epithelial responses and the difference between ingested and circulating chemical species are supported by several lines of evidence. Other claims remain less settled, including consistent meal-driven endotoxemia in humans, the interpretation of indirect barrier markers and the attribution of delayed systemic effects to parent dietary compounds (11, 12, 35, 42). For this reason, the PBEA should be used as a tool for model selection and claim restraint, not as evidence for one fixed pathway from meal intake to inflammatory signaling.

### Limitations of the PBEA model

The PBEA has several limitations. The model does not provide original datasets, mathematical simulations or quantitative transfer values between fractions, and should therefore not be treated as a predictive exposure model. It is a conceptual model intended to organize hypotheses and interpretation, not a validated predictive model. The four fractions should be read as levels of exposure inference rather than fixed biological compartments. In practice, these fractions may overlap, occur at different speeds and be difficult to separate analytically. For example, a plasma metabolite profile in a human study may reflect absorption, enterocyte metabolism, hepatic handling, microbial metabolism, lipoprotein transport and clearance at the same time. Similarly, an epithelial model may show both local intestinal bioactivity and limited transepithelial transfer, while a human plasma study may show absorbed or post-absorptive exposure without proving a tissue-level mechanism.

The evidence base used to build the PBEA is also heterogeneous. Digestion models, epithelial systems, fermentation experiments, metabolite profiling studies and human postprandial trials each answer different questions. They should not be treated as equivalent forms of validation. The model is therefore best used to guide model selection, study design and claim restraint, rather than to force every study into a rigid category or to establish a new causal theory.

At present, the PBEA is likely to be most useful in research on polyphenols, lipids, microbial metabolites, barrier interpretation, endotoxin-related markers and cardiometabolic inflammation, where confusion between intake, exposure and mechanism is common. Its value in other areas of mechanistic nutrition should be judged with the same care and will depend on whether suitable compartment-specific measurements are available.

## Conclusion

Mechanistic nutrition research needs clearer boundaries between intake, luminal release, local intestinal activity, epithelial transfer, microbial transformation and systemic exposure. The PBEA is proposed as a practical model for keeping claims matched to the compartment actually measured. It separates bioaccessibility, intestinal bioactivity, absorption and systemic relevance into distinct levels of exposure inference. This distinction should prevent common overinterpretations such as equating intake with exposure; bioaccessibility with absorption; detection in plasma with physiological relevance; or indirect markers of endotoxin exposure with evidence for altered intestinal permeability. The model does not define a single pathway from meals to inflammation. Its value lies in helping researchers design better studies, choose appropriate models, interpret timing more carefully and keep claims within the evidence generated by postprandial nutrition studies.
